# Tuberculosis Transmission in Households and Classrooms of Adolescent Cases Compared to the Community in China

**DOI:** 10.3390/ijerph15122803

**Published:** 2018-12-10

**Authors:** Dongxiang Pan, Mei Lin, Rushu Lan, Edward A Graviss, Dingwen Lin, Dabin Liang, Xi Long, Huifang Qin, Liwen Huang, Minying Huang, Virasakdi Chongsuvivatwong

**Affiliations:** 1 Department of Tuberculosis Prevention and Control, Guangxi Zhuang Autonomous Region Center for Disease Prevention and Control, Nanning 530021, Guangxi, China; gxpandongxiang@163.com (D.P.); gxlinmei@126.com (M.L.); gxlrshu@163.com (R.L.); drldw@163.com (D.L.); gxmu958@163.com (D.L.); 18778978664@163.com (H.Q.); wenzi0629@126.com (L.H.); hmy9610@163.com (M.H.); 2 Epidemiology Unit, Faculty of Medicine, Prince of Songkla University, Hatyai 90110, Songkhla, Thailand; 3 Department of Pathology and Genomic Medicine, The Center for Molecular and Translational Human Infectious Diseases Research, Houston Methodist Research Institute, Houston, TX 77030, USA; eagraviss@houstonmethodist.org; 4 School of Public Health, Guangxi Medical University, Nanning 530021, Guangxi, China; longxi0805@163.com

**Keywords:** tuberculosis, contact investigation, adolescent, household, classroom

## Abstract

The aim of this paper is to evaluate the link between the history of exposure to tuberculosis (TB) in the household and diagnosed TB cases at school, and to compare the detection rate of active TB among household contacts and classroom contacts of adolescent TB cases with the rates among contacts of healthy controls. From November 2016 to December 2017, a prospective matched case-control study was conducted using passively identified index adolescent student cases from the TB surveillance system and healthy controls (matched by county, school type, sex, age and ethnicity). Contacts in households and classrooms of index cases and of controls were investigated. Matched tabulation of 117 case-control pairs revealed exposure to TB in the household as a strong risk factor (odds ratio (OR) = 21.0, 95% confidence interval (CI): 3.4, 868.6). Forty-five (case detection rate 0.69%) and two (case detection rate 0.03%) new active TB cases were detected among 6512 and 6480 classroom contacts of the index cases and controls, respectively. Having an index case in the classroom significantly increased the risk of classmates contracting active TB (OR = 22.5, 95% CI: 5.9, 191.4). Our findings suggested that previous exposure to TB in the household could lead a child to catch TB at school, then spread TB to classmates.

## 1. Introduction

Childhood and adolescent tuberculosis (TB) continues to be a growing concern and problem in countries with a medium or high prevalence of TB. Worldwide in 2017, the incidence of childhood TB was approximately 1 million with 230,000 deaths [1]. To achieve the World Health Organization (WHO) End TB Strategy, systematic screening of contacts including children will be important [2].

Contact investigation is considered a means to improve early case detection and decrease the transmission of *M tuberculosis (Mtb)* in high-incidence areas [3]. Adolescent active TB case detection rates in school outbreak settings have been reported to be 2.9% in Korea [4], 4.1% in China [5], and 6% in the United Kingdom [6], suggesting TB is actively transmitted in school [5,7,8], particularly in students who share classes with the index case patient [9]. However, in most school TB outbreak investigations, contact investigation is usually confined to the school, while household transmission is often overlooked and neglected [10,11], and where potential household contacts with active disease may be identified. It has been well documented that household exposure to a known TB case is the primary risk factor for TB [12], especially for children [13]. Adolescents, therefore, may acquire TB infection at school [14] or from the household [15,16]. Clearly in China there is a lack of studies comparing the roles of households and of the classroom in adolescent TB transmission.

Moreover, transmission of TB to adolescents also occurs unnoticed in the community [17]. For example in South Africa, 0.33% of new TB cases were detected among adolescent student screening [18]. In contrast, there was no active TB cases detected in either South Korea [19], Shanghai, China [20] nor the United States [21] among TB screening studies of 153, 1106 and 925 students, respectively. In the TB endemic area of Guangxi Zhuang Autonomous Region of China, there is no data on adolescent TB from households and schools versus the community. 

Based on these shortcomings of adolescent TB epidemiologic information in China, the current objective included: (1) to evaluate the link between history of exposure to TB in the household as well as in the classroom and newly diagnosed TB cases at school; (2) to compare the detection rates of active TB among household contacts and classroom contacts of index TB cases with the rates among contacts of healthy controls.

## 2. Methods

### 2.1. Study Setting

The Guangxi Zhuang Autonomous Region (Guangxi), with a population of 50 million, is one of the highest TB burden provinces in China. In 2016, the reported TB incidence was 112 and 55 per 100,000 among the general and adolescent (15–19 years old) populations, respectively. Although the Chinese Ministry of Health had proposed guidelines on investigating close contacts of infectious TB cases in 2008 [22], contact investigations have been inconsistently carried out due to resource limitations and in general the lack of awareness for TB prevention programs in the general public [23]. There were 5 school-related TB outbreaks reported in Guangxi in 2016. Our study period was from November 2016 to December 2017.

### 2.2. Study Design

The first part of the investigation was a case-control study and served as the first objective; the second part of the investigation was contact investigations for contacts of index cases and control classes, and served as the second objective. 

### 2.3. Study Subjects

#### 2.3.1. Ascertaining Adolescent Index Tuberculosis (TB) Cases 

New active pulmonary TB in an adolescent was diagnosed at the county Center for Disease Prevention and Control (CDC) clinics or hospitals and was immediately confirmed and notified to the Guangxi CDC, where the study team led by the first author undertook the contact investigation. An active pulmonary TB case was defined according to recommended WHO diagnosis criteria [24]. Eligible index cases were middle or high school students and were diagnosed with active pulmonary TB. 

#### 2.3.2. Healthy Adolescent Control Selection 

We selected a healthy control from a selected class from a control school using the following details:

(1) One control school was selected by matching school type (e.g., middle/high school, public/private school) with the school of the index case in the same county.
(2) In the selected control school, a class of similar class size as the class size of index case was chosen for investigation. The matched control class must not have reported any TB case.
(3) In the selected control class, one selected student who had without TB history, had the same gender, age (±2 years), ethnicity and school residential status to the index case was chosen as a healthy control.

#### 2.3.3. Investigation of Index Case and Healthy Control

Both the index TB cases and healthy controls were initially interviewed for background information on themes that included the following items: demographic characteristics, socio-economic status (family income per year, head of household education) and TB contact history. 

#### 2.3.4. Identification of Contacts

Contacts of the index TB case were identified according to national guidelines. The classroom contact was referred to those who had direct contact with the index case in the same class, included classmates and teachers of index case [25]. In China, the classmates of a student in the middle and high school usually study in the same classroom throughout the academic year.

Household contacts was defined as people who lived together with the index TB case in the same house, sharing the same housekeeping arrangements and eating together, including family members (parents, sibling) and grandparents. 

For the healthy controls, household and classroom contacts (representative of the general population) were also identified in the same fashion as the index TB case.

#### 2.3.5. Investigation of Contacts

Investigation of household members of the index TB case included a questionnaire, chest radiography, and sputum smear examination when chest radiography displayed abnormal results. 

Investigation of classmates and teachers of index TB cases included a tuberculin skin test (TST) and questionnaire. Those with suspicious symptoms of TB or TST positive who had undergone chest radiography were further evaluated for TB (sputum smear examination) if appropriate. 

Household contacts and classroom contacts who had abnormal chest radiograph results were given a sputum examination with three sputum specimens (night, morning and a spot sputum). Acid fast smears were prepared, stained (Ziehl–Neelsen), and graded according to WHO recommendations. Further diagnostic procedures for active TB followed the diagnostic criteria of the WHO [24].

Household members and classroom contacts of healthy controls were investigated in the same fashion as the index case.

#### 2.3.6. Tuberculin Skin Test (TST)

The TST was provided for all classroom contacts of index TB cases and healthy controls. The TST was performed by the Mantoux method using 0.1 mL (2 tuberculin units) of tuberculin RT-23 (Statens Serum Institute, Copenhagen, Denmark) inoculated intradermally into the volar surface of the forearm with a standard tuberculin syringe. The reaction was read 48–72 h after plantation. 

In China, the Bacillus Calmette–Guérin (BCG) vaccination has been provided to all newborns for more than half a century. Because of the high BCG vaccination rate, Chinese guidelines consider TST in student as positive if the induration diameter is 15 mm or greater [26].

#### 2.3.7. Questionnaire Interview for Contacts

Household and classroom contacts were asked about whether they had been diagnosed with TB or currently had active TB, and whether they were having any suspicious symptoms (cough of 2 weeks, fever, hemoptysis). A subject with any suspicious history or symptom underwent further investigation for TB. 

#### 2.3.8. Ethics Approval 

The study was approved by the Research Ethics Committee of Prince of Songkla University (No. 59-247-18-5) and Ethical Review Committee of Guangxi (No. GX IRB2016-0049). Before enrollment, consent from the parents/guardians of participants less than 18 years of age was requested and received. Older participants used their own judgment in giving consent.

### 2.4. Statistical Analysis 

**Figure 1:**
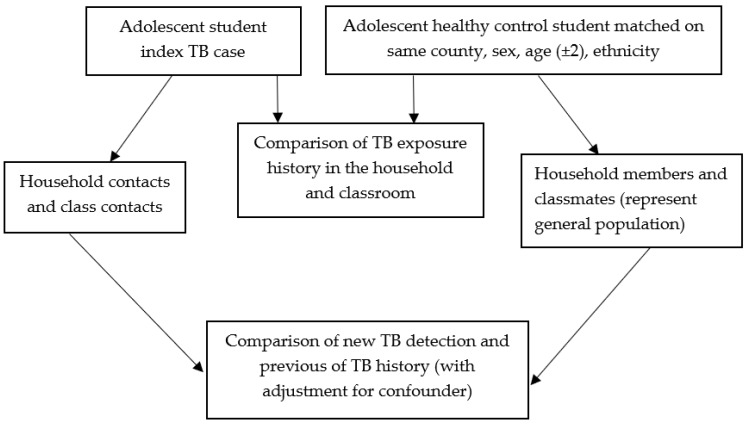
The framework of data analysis.

The analysis followed the framework described in Figure 1. There were two units of analyses levels. The first level of comparison was the adolescent index TB case versus the healthy control matched pairs on various risk factors exposure. Matched odds ratio was calculated based on comparison of the discordant pairs [27]. The second level of comparison was detection rate of active TB and prevalence of previous TB among contacts of the TB index cases and of the healthy controls in the household and classroom. Logistical regression was carried out with adjustment for confounding. The significance level was 0.05. Analysis was carried out using the statistical software package R (version 3.4.2, Vienna, Austria).

## 3. Results

### 3.1. Characteristics of Adolescent TB Index Cases and Healthy Controls

A total of 117 TB cases and 117 healthy controls were recruited. The age of the subjects ranged from 12 to 20, and the mean age ± SD was 16.7 ± 1.9 years. 

Table 1 summarizes the distribution of socio-economic characteristics which were generally balanced between TB cases and healthy controls, except education of the household head which was higher among the healthy control group. Note, that the vast majority (94%) of the index cases were boarding students and the same proportion was selected in the control group.

### 3.2. Comparison of TB Exposure History between the Index Case and the Matched Healthy Control

Table 2 and Table 3 summarize the relationship between TB exposure history in the household and classroom of index TB cases and those of the matched healthy controls, based on the matched analyses of 117 pairs. We identified (in Table 2) 21 matched pairs where the index TB case had a history of previous TB exposure in the household and the healthy control did not. In contrast, only one matched pair was identified where the control had a history of previous TB exposure in the household while the index TB case did not. The risk of index case exposed to previous TB in the household was increased 21.0 (95% confidence interval (CI): 3.4, 868.6) times than the matched healthy control.

A similar analysis, with results shown in Table 3, revealed that the odds ratio (OR) of index case exposed to previous TB in the classroom was 0.8 (0.2, 3.0). Although a higher proportion of index cases had current active TB contact history in the classroom than the controls (12.0% versus 1.7%), the risk of being an index case from exposure to previous and current TB in the classroom was not significantly different (OR = 2.14, 95% CI: 0.87, 5.26) (Table 3).

### 3.3. Results of Contact Investigation to Detect New TB in the Household and Classroom 

Table 4 summarizes the results of the contact investigation in the household and classroom of adolescent index TB cases and healthy controls.

In the household contact investigation, 200 and 199 household contacts were identified from the index TB cases and of the healthy controls, respectively. There were no new active TB patients detected in either group. 

In the classroom contact investigation, of 223 (3.42%) students who were TST positive, 45 (case detection rate 0.69%) new TB cases were detected among 6512 classroom contacts of the index TB cases. Nine of the 45 new TB cases had sputum smear positive TB, and 36 were diagnosed by chest radiography. These cases were from 14 different classes from different schools with the number of new TB cases detected in each class being: 10, 9, 7, 3, 3, 3, 2, 2, 1, 1, 1, 1, 1, and 1. For the healthy controls, among 6480 classroom contacts screened, 87 students (1.34%) were TST positive, of whom 2 (case detection rate 0.03%) new active TB patients (1 who was smear-positive) were identified from 2 different schools. Having an index TB case in the classroom significantly increased odds of classmates contracting active TB by 22.5 times (95% CI: 5.9, 191.4) compared to students without active TB in the classroom.

We also screened the 151 teachers of index TB cases and 120 teachers of healthy controls. There was neither active TB nor a previous history of TB among them.

### 3.4. Comparison of Previous TB among Contacts of Index Cases and Healthy Controls

In the bottom part of Table 4, there were 34 (17%) contacts of index TB cases having a previous TB history in the household. The corresponding number was 3 (1.5%) for healthy control household members. In the classrooms, 5 (0.08%) contacts of index TB cases had a previous TB history; the corresponding number was 6 (0.09%) for healthy control classroom contacts.

For contacts of index cases, the prevalence of previous TB among the household contacts was significantly higher than that of the classroom contacts (OR = 265.5, 95% CI: 101.5, 895.1). The difference was also significant among household contact and classroom contacts of the healthy controls (OR = 16.5, 95% CI: 2.7, 77.8).

### 3.5. Multivariable Model Assessed Contacts with Previous TB History in the Household and Classroom

We ran logistic regression to predict previous TB, having age, sex, and type of contacts (household versus classroom) and contacts of subjects (contacts of index case versus contacts of control) as independent variables (not shown). The conclusion of the multivariate was similar to the bivariate analysis. The odds ratio (95% CI) of previously having TB in the household was 247.8 (65.9, 931.8) among index TB case contacts and 16.4 (3.0, 88.9) among contacts of the healthy controls. 

## 4. Discussion

TB exposure history in the household but not in the classroom was a significant risk factor for being an adolescent student index TB case. The new detection cases among classroom contacts of the index TB cases was 45 and 2 in the control classes, a 22.5-fold increase in odds compared the contacts of healthy controls. Prevalence of previous TB was more common in the household contacts than in the classrooms in both index cases and healthy controls. 

After carefully matching controls, our finding reflected that previous TB exposure history in the household increases the odds of adolescent students contracting active TB in school 21-fold. This conclusion is consistent with other previous case-control studies in children in Brazil [28] and rural Africa [29]. 

From contact investigations, there were 45 new TB (case detection rate 0.69%) cases diagnosed in the classes of index cases, which suggested that the classroom was an important place of TB transmission. In recent years, multiple studies have reported TB outbreaks in Chinese middle schools and high schools [5,30,31,32,33], which highlighted adolescent TB as a growing problem. Other countries have also reported school TB outbreaks, such as in the United Kingdom, Italy, Israel, United States, France and Korea [6,8,9,34,35,36,37,38], indicating that classroom exposure increases the risk of acquiring active TB. The value of odds ratio was not available in those reports, as they did not have a case detection rate in a control population. 

We did not find any new TB cases at the household level. Our index TB cases were mostly (94%) boarding students, thus they have more proximity to their peers than to their household members [39,40]. Nevertheless, despite the low detection rates found in the household contacts, contact tracing in households should not be ignored. In fact, our zero detection rate in 200 household members has 95% CI (0%, 1.83%). This fraction is not significantly different from the detection of the 1 of 40 household contact in Swaziland [10] (*p*-value = 0.167 by Fisher Exact testing). Moreover, a report from United States detected one genito-urinary TB case among five household contacts from a student during a TB contact investigation [11].

The case detection rate among contacts of our control was not negligible (0.03%). This rate was higher than the rate in a school survey in an affluent city of China (Shanghai) [20] and in a high-income country like the U.S. [11]. However, it was 10 times lower than the result from a school screening in South Africa (0.3%) [18], where TB/human immunodeficiency virus (HIV) is highly prevalent. Thus in middle-income countries, sporadic adolescent TB transmission can occur unnoticed in the school until an outbreak situation occurs. 

Finally, the linkage between TB exposure in the household and an outbreak in the classroom could be suggested by our research. Index TB cases had household members with a history of TB as a very strong risk factor (OR = 21.0). These index cases led to the detection of more new TB cases in the classroom than in the population (OR = 22.5). Thus, TB might have been transmitted from the household member to an adolescent who subsequently became active with TB at school and caused the school transmission. This should have been prevented by careful contact tracing and contact management in the past. Unfortunately, contact tracing has not been done properly in China until recently [5,23,31].

One limitation of this study was lack of genetic information of *Mtb* among classmate TB and those from household members with previous TB. Secondly, there is a lack of information as to how often boarding students return home for short visits where additional “unknown” exposure may have taken place. Thirdly, we used the TST screening method which has only a moderately high sensitivity and specificity. Despite these limitations, our data has highlighted the need to improve TB contact tracing and intervention in the household and school. This could in turn diagnose TB in earlier stages and reduce the subsequent risk of school TB outbreak. 

## 5. Conclusions

Inadequate household contact investigation and management in the past may have left infected children untreated. These children then could subsequently become active TB cases and transmit TB in the classroom. 

## Figures and Tables

**Table 1:** Characteristics of adolescent index tuberculosis (TB) case and healthy control.

Variables	Index TB (*n* = 117)		Healthy Control (*n* = 117)	
	*n*	%	*n*	%
**Age, Year**				
12–15	32	27.35	31	26.5
16–19	85	72.65	86	73.5
**Gender**				
Female	63	53.85	63	53.85
Male	54	46.15	54	46.15
**Ethnicity**				
Others	54	46.15	54	46.15
Han	63	53.85	63	53.85
**Head of household education**				
Primary school	30	25.64	13	11.11
Middle school	83	70.94	101	86.32
Collage	4	3.42	3	2.56
**Family income per year (yuan)**				
<10,000	29	24.79	17	14.53
10,000–30,000	52	44.44	46	39.32
30,001–60,000	26	22.22	39	33.33
>60,000	10	8.55	15	12.82
**School type**				
Middle school	46	39.32	46	39.32
High school	71	60.68	71	60.68
**School residential**				
Ambulatory	7	5.98	7	5.98
Boarding	110	94.02	110	94.02

**Table 2:** Exposed to previous TB in household of index case and the matched healthy control (117 matched pairs).

Variable	Status of Exposure of Index Case	
	No	Yes
**Exposed to previous TB in the household**		
Status of exposure of the matched healthy control		
No	93	21
Yes	1	2
Odds ratio (OR) (95% confidence interval (CI))	21.0 (3.4–868.6)	

**Table 3:** Exposed to previous/current TB in classroom of index case and the matched healthy control (117 matched pairs).

Variable	Status of Exposure of Index Case	
	No	Yes
**Exposed to previous TB in the classroom**		
Status of exposure of the matched healthy control		
No	107	4
Yes	5	1
OR (95% CI)	0.8 (0.2, 3.0)	
**Exposed to TB in the classroom (included previous and current TB)**		
Status of exposure of the matched healthy control		
No	94	15
Yes	7	1
OR (95% CI)	2.14 (0.87, 5.26)	

**Table 4:** New active TB and previous TB among contacts of index case and healthy control.

Outcome Variables	Contacts of Index Cases (*n* (%))		OR (95% CI)	Healthy Contacts of Controls (*n* (%))		OR (95% CI)
	In Household	In Classroom		In Household	In Classroom	
**New active TB**						
No	200 (100.00)	6467 (99.31)		199 (100.00)	6478 (99.97)	
Yes	0	45 (0.69)	-	0	2 (0.03)	-
**Previous TB**						
No	166 (83.0)	6507 (99.92)		196 (98.49)	6474 (99.91)	
Yes	34 (17.0)	5 (0.08)	265.5 (101.5, 895.1)	3 (1.51)	6 (0.09)	16.5 (2.7, 77.8)
